# An Assessment of Anti-Ganglioside Antibodies for the Diagnosis of Avian Ganglioneuritis and Potential Correlation with Gross Lesions, Microscopic Findings and Serologic Titers in Cockatiels Challenged with Parrot Bornavirus

**DOI:** 10.3390/ani16101466

**Published:** 2026-05-10

**Authors:** Bianca Bücking, Anna Maria Gartner, Sibylle Herzog, Christiane Herden, Julia Heiker, Anne Schmidt, Kathrin Büttner, Michael Lierz

**Affiliations:** 1 Clinic for Birds, Reptiles, Amphibians and Fish, Justus Liebig University Giessen, Frankfurter Str. 114, 35392 Giessen, Germany; 2 Institute of Virology, Justus Liebig University Giessen, Schubertstr. 81, 35392 Giessen, Germany; 3 Institute for Veterinary Pathology, Justus Liebig University Giessen, Frankfurter Str. 96, 35392 Giessen, Germany; 4 Unit for Biomathematics and Data Processing, Faculty of Veterinary Medicine, Justus Liebig University Giessen, Frankfurter Str. 95, 35392 Giessen, Germany

**Keywords:** proventricular dilatation, immunopathogenesis, ganglioside, anti-ganglioside antibodies, avian ganglioneuritis, parrot bornavirus

## Abstract

Avian ganglioneuritis is one of the most widespread diseases of psittacines and is caused by parrot bornavirus (PaBV). It has been suggested that PaBV causes a T-cell-mediated immunopathology comparable to Borna disease virus 1 (BoDV-1). However, the factors involved in progression from infection to clinical disease in psittacines remain poorly understood. It has been proposed that autoantibodies directed against endogenous gangliosides of the central and peripheral nervous systems contribute to disease development. These anti-ganglioside antibodies have been suggested as potential diagnostic markers for birds developing clinical avian ganglioneuritis independent from the causing infectious agent. In this study, 257 plasma samples from cockatiels (*Nymphicus hollandicus*) experimentally infected with PaBV, with known infection status as well as defined clinical and postmortem outcomes, were submitted in a blinded manner to a commercial laboratory for anti-ganglioside antibody testing. The results were compared with the presence of anti-PaBV antibodies, clinical signs, gross pathological findings, and histopathological results. Among 25 birds with dilated proventriculi at the time of necropsy, 9 (36%) tested positive for anti-ganglioside antibodies, whereas 16 (64%) tested negative. Of 56 birds with histopathological avian ganglioneuritis, 26 (46.43%) tested positive and 30 (53.57%) tested negative. Among 33 birds without histopathological inflammatory lesions, 19 (57.58%) tested negative and 14 (42.42%) tested positive. These results indicate that no association between the occurrence of avian ganglioneuritis and the detection of anti-ganglioside antibodies was seen.

## Simple Summary

Avian ganglioneuritis is one of the most widespread diseases of psittacines and is caused by parrot bornavirus (PaBV). The detection of anti-ganglioside antibodies has been proposed as a simple diagnostic tool to differentiate between birds infected with PaBV and those developing clinical avian ganglioneuritis. In this study, 257 plasma samples from cockatiels experimentally infected with PaBV, with known infection status as well as defined clinical and postmortem outcomes, were submitted in a blinded manner to a commercial laboratory for anti-ganglioside antibody testing. The results were compared with the detection of anti-PaBV antibodies, clinical signs, and gross pathological and histopathological findings. No association was found between the occurrence of avian ganglioneuritis and the detection of anti-ganglioside antibodies.

## 1. Introduction

Proventricular dilatation disease (PDD) has been described since 1970 [1,2] and was initially referred to as macaw wasting syndrome [3]. In subsequent years, proventricular dilatation disease has been reported in more than 50 psittacine species, predominantly affecting adult birds [1]. Clinical signs include nonspecific findings such as apathy, weakness, and ruffled feathers, as well as typical gastrointestinal signs including regurgitation, diarrhea with undigested seeds, weight loss, and emaciation [4,5]. Central nervous system (CNS) signs such as ataxia, tremors, seizures, blindness, and sudden death have been reported [6]. Histopathologically, PDD is characterized by nonsuppurative mononuclear inflammation of peripheral neural ganglia, predominantly composed of lymphocytes, macrophages and plasma cells. Lesions affect the peripheral nerves and ganglia, particularly within the gastrointestinal tract, but also involve the CNS [7,8,9]. This inflammation leads to muscle atrophy and impaired motility, resulting in an accumulation of undigested food and subsequent dilatation of the ventriculus, proventriculus, and intestines [10]. The etiology remained unknown for many years despite a presumed infectious cause. In 2008, two independent research groups identified a novel *Orthobornavirus* in parrots affected by PDD [11,12]. Subsequent experimental infection studies confirmed orthobornaviruses as an etiological agent of PDD in various psittacine species [13,14,15,16,17]. Natural infection with parrot bornavirus and subsequent development of avian ganglioneuritis has been demonstrated in a wide variety of psittacine species [18]. Horizontal transmission is presumed to occur via the fecal–oral route [19]. However, oronasal or peroral routes of infection could not be proven in infection trials [20]. Field observations indicate limited horizontal transmissibility, as persistently infected birds remain in close contact with uninfected individuals for extended periods without transmission [13,21,22,23,24]. Heckmann et al. [25] suggested that horizontal infection occurs via wounds, likely spreading to the spinal cord and brain, followed by centrifugal spread into peripheral nerves and ganglia [26]. Vertical transmission of avian orthobornaviruses has long been hypothesized [27,28] and was demonstrated by Link et al. [29] in cockatiels. However, because not all infected birds develop clinical disease [18,30,31], the factors contributing to progression from infection to clinical disease are poorly understood. It has been proposed that similar to mammalian bornaviruses, PaBV (parrot bornavirus) infections induce an immune-mediated pathology. For Borna disease virus 1, a CD4+ and CD8+ T-cell-mediated immunopathology has been demonstrated [32]. For parrot bornaviruses, Hameed at al. [33] showed that treatment with cyclosporin A, a T-cell inhibitor, prevented inflammatory lesions and the onset of clinical symptoms in cockatiels infected with PaBV-2. This supports an immunopathological mechanism comparable to that observed in mammals infected with Borna disease virus 1 [33]. Gartner et al. [34] demonstrated that the age of the bird at the time of infection influences disease development. In this trial, 11 adult cockatiels and 11 immunologically immature cockatiel nestlings between 1 and 6 days of age were infected intravenously with PaBV-4. While nine adult cockatiels developed clinical signs of avian ganglioneuritis, none of the juvenile birds did, despite showing milder inflammatory lesions [34]. The authors suggested that the immature immune system of young birds limits clinical disease and concluded that these findings support an immune-mediated pathogenesis.

Other authors have proposed that avian ganglioneuritis is caused by the development of autoantibodies against neuronal gangliosides of the nervous system, similar to Guillain–Barré syndrome in humans [35]. Such antibodies can be induced by various infections and are not restricted to PaBV infections. Other pathogens (e.g., *Campylobacter jejuni* or *Escherichia coli*) may also play a role [36,37]. It has been reported that up to 98% of psittacine birds showing typical symptoms and histological lesions of avian ganglioneuritis develop high anti-ganglioside antibody titers [38,39], suggesting a correlation between anti-ganglioside antibody presence and clinical disease. Rossi et al. [40,41] experimentally inoculated six cockatiels (*Nymphicus hollandicus*) with purified gangliosides combined with adjuvant administered perorally or intraperitoneally. Following booster administration after one month, all of the intraperitoneally inoculated birds and 33% of the perorally inoculated group showed typical neurological and gastrointestinal signs. Four of the six cockatiels showed typical lymphoplasmacytic ganglioneuritis in crop biopsies [40,41]. In another study, 11 White Leghorn chickens and 2 Quaker parrots (*Myiopsitta monachus*) received purified gangliosides with adjuvant treatment via repeated intramuscular injections: twice within 31 days for the chickens and three times at 24-day intervals for the Quaker parrots. Two additional Quaker parrots received crude nervous extracts. Six chickens and one Quaker parrot served as negative controls without any injections. While only one of the chickens developed difficulty walking (later attributed to extensive granulomatous steatitis and myositis at the injection site), three of four Quaker parrots showed nonspecific clinical signs (depression, weight loss) but no PDD-typical gastrointestinal or neurological signs. The inoculated Quaker parrots developed anti-ganglioside antibodies but no PDD-typical myenteric ganglioneuritis. The authors concluded that anti-ganglioside antibodies are not involved in the development of avian ganglioneuritis [42].

Based on the hypothesis of anti-ganglioside antibodies, a clinical test for the detection of these antibodies in psittacines is commercially available. The presence of anti-ganglioside antibodies has been proposed as an indicator of avian ganglioneuritis independent of the underlying cause.

Given the incomplete understanding of the pathological mechanisms underlying avian ganglioneuritis following PaBV infection, proper diagnosis of the disease still poses an enormous diagnostic challenge. This is furthermore impeded by intermittent shedding or asymptomatic carriers [30,31]. Therefore, positive molecular or serological test results for PaBV in clinically ill birds do not necessarily identify the virus as the cause of the symptoms. Differentiating between PaBV-infected birds with unrelated clinical conditions and those with PaBV-related avian ganglioneuritis remains difficult. Additional diagnostics are usually necessary to exclude other possible causes of the symptoms. A clinical test that directly detects avian ganglioneuritis in diseased birds would therefore be advantageous. If anti-ganglioside antibodies are key factors for the induction of ganglioneuritis, a serological test to detect them might fill this gap.

Therefore, this study investigated whether the detection of anti-ganglioside antibodies can be used to identify avian ganglioneuritis. For this purpose, birds with known clinical and histopathological outcomes, as well as known PaBV infection status as one of the proven causes of avian ganglioneuritis, were used.

## 2. Materials and Methods

### 2.1. Birds and Samples

In total, 257 plasma samples were used in this study. These samples originated from 91 cockatiels from previous infection studies [16,17,19,24,34] (see Table 1). In those studies, cockatiels were experimentally infected with PaBV-2 or -4 (parrot Bornavirus 2 or 4), and plasma samples were collected prior to infection or at various time points during the trial. All birds originated from a specific pathogen-free flock that had repeatedly tested negative for *Chlamydia psittaci*, psittacine herpesvirus, avian paramyxovirus-1, and PaBV.

The cockatiels were monitored for 174 to 233 days after virus inoculation, depending on the respective study. Blood samples were collected once weekly for the detection of anti-PaBV antibodies. If a bird died or was euthanized because of severe clinical signs, or reached the end of each study, a full postmortem examination was performed, and samples of various organs (see below) were taken for histopathology and PaBV RNA detection. Consequently, for each sample used in this study, the anti-PaBV antibody titer and clinical appearance of the bird were known. For samples collected at the end of this study, histopathological findings and the presence of viral RNA at the time of sampling were also available.

The following samples were selected from previous studies:

### 2.2. Indirect Immunofluorescence Assay (iIFA) for Detection of Anti-PaBV Antibodies

An indirect immunofluorescence assay for the detection of specific antibodies against PaBV (parrot bornavirus) was performed using persistently PaBV-infected CEC-32 cells, as previously described [43]. For statistical analysis and comparison with the anti-ganglioside antibody detection assay, the samples were grouped according to their PaBV antibody titers. Titers of <1:10 were assigned to titer group 0, 1:10–1:80 to titer group 1, 1:160–1:640 to titer group 2, 1:1280–1:2560 to titer group 3, and >1:2560 to titer group 4.

### 2.3. Necropsy

**Figure 1:**
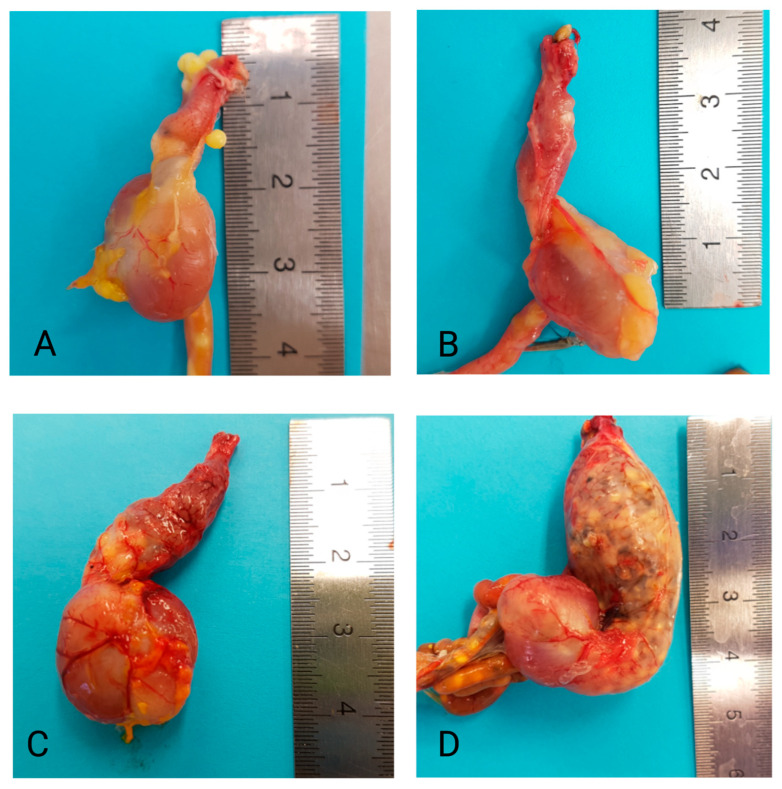
Representative pictures of proventricular dilatation (0 = no dilatation (**A**), 1 = mild dilatation (**B**), 2 = moderate dilatation (**C**), and 3 = severe dilatation (**D**)). The pictures were obtained from the trial of Gartner et al. [34].

Necropsy was performed as described in the studies from which the samples originated [16,17,20,25,34]. In short, macroscopic alterations of the proventriculus were evaluated using a scoring system: 0 = no dilatation, 1 = mild dilatation, 2 = moderate dilatation, and 3 = severe dilatation (see Figure 1). Samples were collected from the cerebrum, cerebellum, spinal cord, eye, sciatic nerve, heart, liver, kidney, adrenal gland, spleen, crop, proventriculus, ventriculus, small and large intestines, pancreas, pectoral muscle, gonads, and skin with feathers, and were stored at −80 °C; an identical set of samples were fixed in 10% buffered formalin for histopathological examination. As described in the original studies, for bacteriological examination, samples from the heart, liver, and lungs were collected and cultured on sheep blood agar and Gassner lactose agar (Water-Blue Metachrome Yellow Lactose Agar; Oxoid Deutschland GmbH, Wesel, Germany) for 48 h at 37 °C. Samples from the crop and proventriculus were cultured on Kimmig fungal agar (Oxoid Deutschland GmbH) for 5 days at 28 °C. Smears from the crop and proventriculus were stained with Giemsa and examined for yeasts. Direct smears from intestinal contents were examined for parasites.

### 2.4. Histopathology

**Figure 2:**
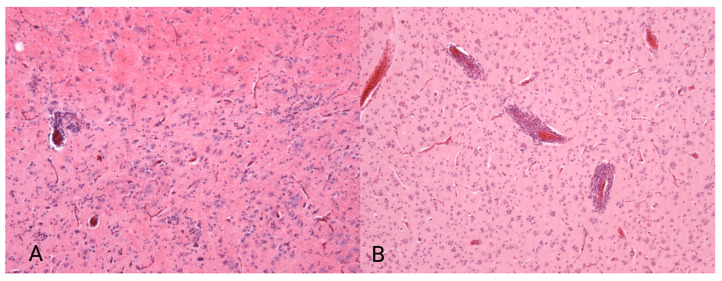
Representative pictures of mild (**A**) and severe (**B**) perivascular cuffing in the brain. The pictures were obtained from the trial of Gartner et al. [34].

Histopathology was performed as described in the original studies from which the samples were obtained [16,17,20,25,34]. Briefly, hematoxylin and eosin staining was performed to identify inflammatory infiltrates or other pathological alterations in the examined organs. Scoring was based on the detection of typical mononuclear infiltrations: 0 = no mononuclear infiltrates observed, 1 = mild mononuclear infiltrates, 2 = moderate mononuclear infiltrates, and 3 = severe mononuclear infiltrates (see Figure 2).

### 2.5. Anti-Ganglioside Antibody Testing

The plasma samples were sent, blinded, to a diagnostic laboratory that offers anti-ganglioside antibody testing for birds using an enzyme-linked immunosorbent assay. The results were reported by the laboratory as Absorptance (405 nm) and divided into different titer groups. The laboratory also provided an interpretation guideline, which was used in this study. Results under 160 were considered negative (group 0), those up to 180 were labeled “developing titer” (group 1), those up to 220 were labeled “mild clinical disease” (group 2), those over 220 were considered “chronic disease” (group 3), and those over 315 were considered “advanced clinical disease” (group 4).

### 2.6. Statistics

The exact Pearson chi-square test was used to compare clinical data, histopathological inflammatory lesions, and gross pathological changes (dilatation of the proventriculus) with the detection of anti-ganglioside antibodies. To assess the association between the detection of anti-PaBV antibodies and the detection of anti-ganglioside antibodies, the exact Pearson chi-square test with Monte Carlo simulation was used. All 257 plasma samples were included in the analyses of clinical data and anti-PaBV antibodies, whereas only 89 plasma samples obtained at euthanasia or immediately prior were used for analyses involving gross pathology and histopathology. Statistical analyses were conducted using SAS version 9.4 (SAS Institute Inc., Cary, NC, USA) [44].

## 3. Results

### 3.1. Anti-PaBV and Anti-Ganglioside Antibodies

**Figure 3:**
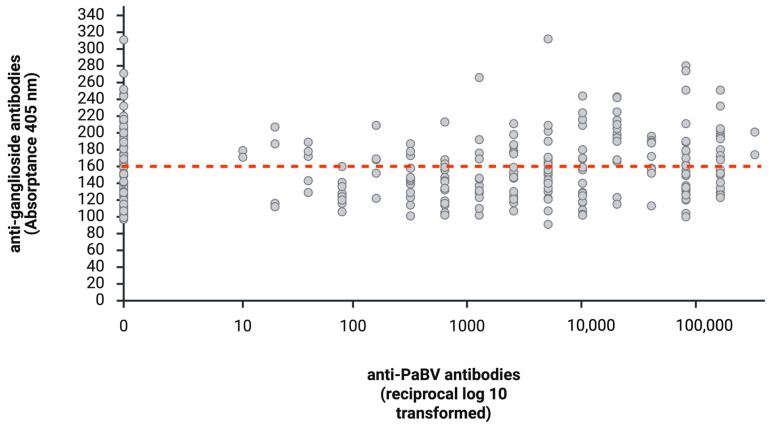
Distribution of anti-ganglioside antibodies (Absorptance 405 nm) and anti-PaBV antibodies (reciprocal). Samples of <160 are considered negative and those of >160 are considered positive in anti-ganglioside antibody testing (red dotted line). (Created with BioRender.com).

Of the 257 plasma samples included in this study, 62 (24.12%) had no detectable anti-PaBV antibodies. Of these 62 samples, 36 (58.06%) tested negative for anti-ganglioside antibodies and 26/62 (41.98%) tested positive. A total of 195/257 plasma samples (75.88%) showed detectable anti-PaBV antibodies. Among these 195 samples, 114/195 (58.46%) tested negative for anti-ganglioside antibodies and 81/195 (41.54%) tested positive (see Table 2 and Figure 3).

**Figure 4:**
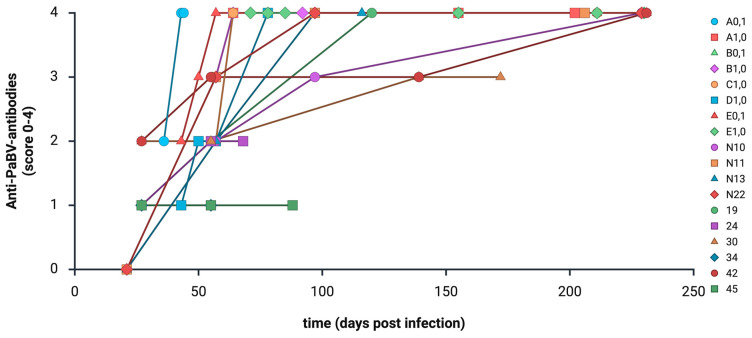
The development of anti-PaBV antibodies (score 1–4; see Table 1) in 18 birds with clinical symptoms during the trial and histopathological inflammatory lesions at the end of the trial. (Created with BioRender.com).

**Figure 5:**
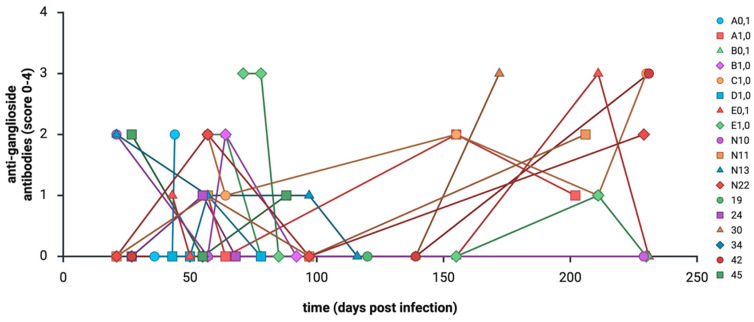
The development of anti-ganglioside antibodies (score 0–4; see Table 1) in 18 birds with clinical symptoms during the trial and histopathological inflammatory lesions at the end of the trial. (Created with BioRender.com).

**Figure 6:**
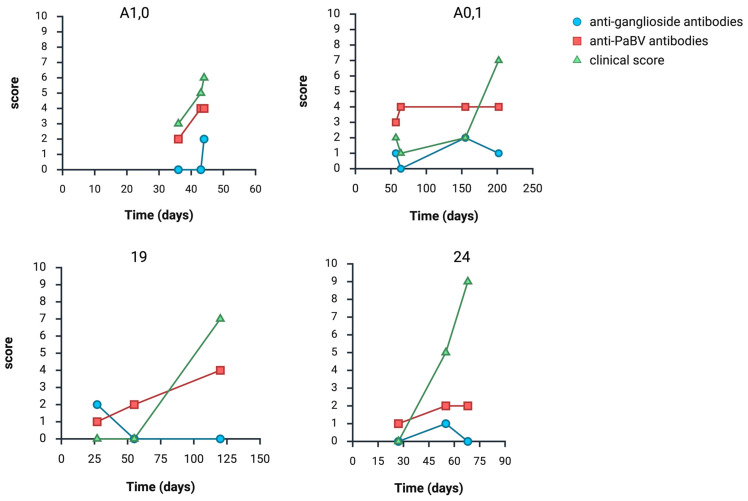
Exemplary longitudinal antibody development (score 0–4; see Table 1) of four birds with clinical signs (score 0–10) and histopathological inflammatory lesions at the end of the trial. (Created with BioRender.com).

As previously described [16,17,34], all experimentally PaBV-infected birds developed anti-PaBV antibodies and reached a plateau. By contrast, anti-ganglioside antibody levels showed considerable longitudinal fluctuations during the trials (see Figure 4, Figure 5 and Figure 6).

The exact Pearson chi-square test with Monte Carlo simulation was used to assess the association between anti-PaBV antibody detection and anti-ganglioside antibody detection. No significant association was found between the detection of anti-ganglioside and anti-PaBV antibodies (*p* = 0.3884).

### 3.2. Clinical Signs and Detection of Anti-Ganglioside Antibodies

A total of 33 out of 257 samples (12.84%) were collected from animals that showed clinical symptoms of avian ganglioneuritis on the day of collection. Of these, 18/33 (54.55%) tested negative (<160 nm; group 0), whereas 15/33 (45.45%) tested positive for anti-ganglioside antibodies. In total, 224/257 samples (87.16%) were collected from animals with no clinical signs of avian ganglioneuritis. Of these, 132/224 (58.93%) tested negative (<160 nm; group 0) and 92/224 (41.07%) tested positive for anti-ganglioside antibodies. The exact Pearson chi-square test was used to assess the association between clinical signs and the detection of anti-ganglioside antibodies. No significant association was found (*p* = 0.4345).

### 3.3. Gross Pathology Lesions and Detection of Anti-Ganglioside Antibodies

A total of 25/89 animals (28.09%) showed macroscopic alterations on gross examination of the proventriculus at the time of sampling. Of these, 6/89 (6.74%) showed mild dilatation of the proventriculus, 10/89 (11.24%) showed moderate dilatation, and 9/89 (10.11%) showed severe dilatation. Among these 25 animals, 9 (36%) showed detectable anti-ganglioside antibody titers, whereas 16 (64%) tested negative. Among the 6 animals with mild dilatation, 3 (50%) tested positive for anti-ganglioside antibodies and 3 (50%) tested negative. Of the 10 birds with moderate dilatation of the proventriculus, 7 tested negative and 3 (30%) tested positive. Of the nine birds depicting severe dilatation, six (66.67%) tested negative and three (33.33%) tested positive for anti-ganglioside antibodies. A total of 64/89 animals (71.91%) showed no macroscopic alterations on gross pathology. Of these, 33/64 (51.56%) tested negative and 31/64 (48.44%) tested positive for anti-ganglioside antibodies. For more details, see Figure 4.

**Figure 7:**
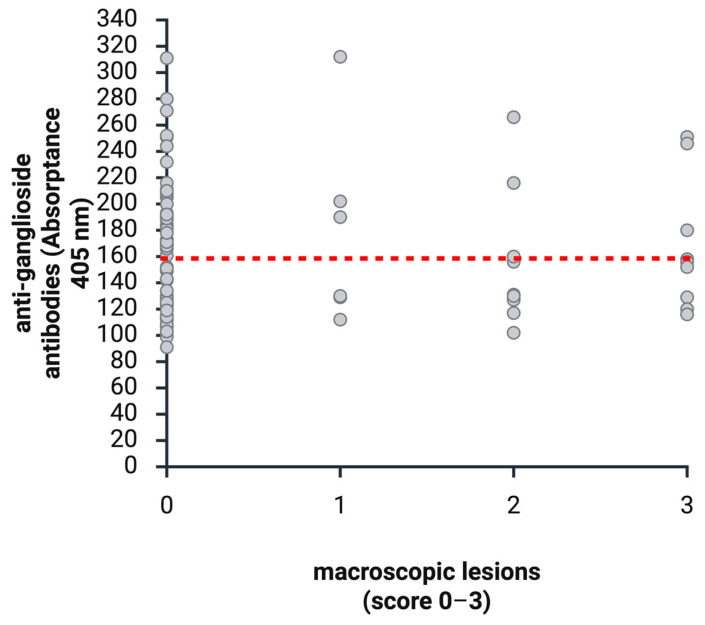
Anti-ganglioside antibodies and macroscopic lesions of the proventriculus (0 = no dilatation, 1 = mild dilatation, 2 = moderate dilatation, and 3 = severe dilatation). Samples < 160 are considered negative and >160 positive in anti-ganglioside antibody testing (red dotted line). (Created with BioRender.com).

The Pearson chi-square test was used to assess the association between anti-ganglioside antibodies and the presence of dilatation of the proventriculus. No significant association was found (*p* = 0.2912) (see Figure 7).

### 3.4. Histopathological Lesions and Detection of Anti-Ganglioside Antibodies

**Figure 8:**
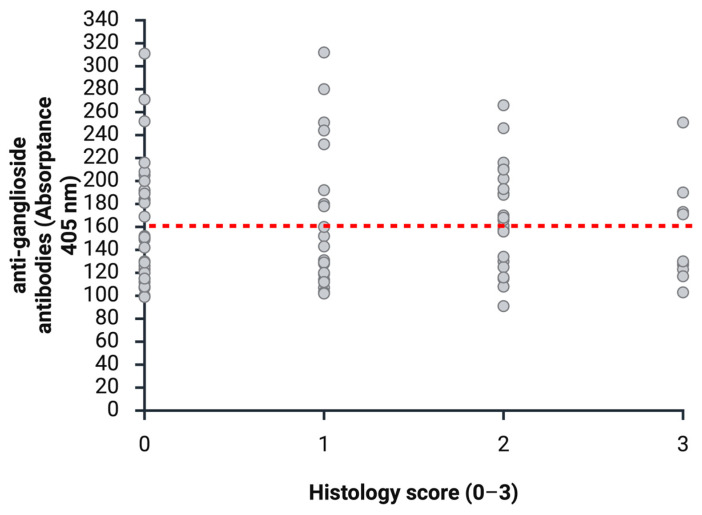
The distribution of anti-ganglioside antibody levels in relation to histologic lesions. Samples < 160 are considered negative and >160 positive in anti-ganglioside antibody testing (red dotted line). Histologic lesion scores: 0 = no mononuclear infiltrates visible, 1 = mild mononuclear infiltrates, 2 = moderate mononuclear infiltrates, and 3 = severe mononuclear infiltrates. (Created with BioRender.com).

Of 89 birds, 33 (37.08%) exhibited no mononuclear inflammatory lesions. Of these, 19/33 (57.58%) tested negative and 14/33 (42.42%) tested positive for anti-ganglioside antibodies (see Figure 5). A total of 56/89 birds (62.92%) showed mononuclear inflammatory nerval lesions at the time of sampling. Of these 56 birds, 30 (53.57%) tested negative and 26 (46.43%) tested positive for anti-ganglioside antibodies. Among these 56 birds with mononuclear inflammatory lesions, 22 (39.29%) showed mild infiltrates. Of these, 8/22 (36.36%) tested positive for anti-ganglioside antibodies, whereas 14/22 (63.64%) tested negative. A total of 24/56 (42.86%) birds showed moderate mononuclear inflammatory lesions. Of these, 10/24 (41.67%) tested negative and 14/24 (58.33%) tested positive. A total of 10/56 (17.86%) birds showed severe mononuclear inflammatory lesions; 6/10 (60%) tested negative and 4/10 (40%) tested positive for anti-ganglioside antibodies. The exact Pearson chi-square test was used to assess the association between mononuclear inflammatory lesions and anti-ganglioside antibody detection. No significant association was found (*p* = 0.2069) (see Figure 8).

## 4. Discussion

PDD (proventricular dilatation disease) is a major concern in psittacine medicine. It affects parrots worldwide and remains a threat to major breeding programs, including those important for species conservation. When PaBV (parrot bornavirus) is identified as the causative agent of clinical disease, flock management strategies aim to eliminate the virus from affected collections [45]. However, many PaBV-positive birds remain clinically healthy [30,31,45], complicating diagnosis. Birds with neurological or gastrointestinal signs may test positive for PaBV despite alternative causes. Therefore, a diagnostic test that reliably identifies PDD-associated inflammatory lesions would be valuable. Detection of anti-ganglioside antibodies has been proposed as a potential diagnostic tool for avian ganglioneuritis [37,38,39,40,41,43]. To evaluate this hypothesis, plasma samples obtained from experimental PaBV infection trials were analyzed for anti-ganglioside antibodies. This study specifically assesses their diagnostic value rather than their pathogenic role. These samples originated from birds with known PaBV infection status, as a proven cause of PDD, and concurrent clinical signs. For a subset of samples, corresponding gross and histopathological status was available because samples were collected at or shortly before the time of death.

For the first time, samples derived from birds in experimental PaBV infection trials were used to assess anti-ganglioside antibodies. This enabled the inclusion of well-characterized samples with known infection, clinical, and histopathological status, including longitudinal samples from individual birds. In contrast, such detailed information is typically unavailable in clinical cases, complicating interpretation.

In general, no association was found between the production of anti-PaBV antibodies and anti-ganglioside antibodies. Previous infection studies demonstrated consistent increases in anti-PaBV antibody titers in all infected birds [16,17,34], whereas no comparable pattern was detected for anti-ganglioside antibodies in the present cohorts. A total of 195/257 samples (75.88%) showed detectable anti-PaBV antibodies. Of these, 114/195 (58.46%) tested negative for anti-ganglioside antibodies, and 81/195 (41.54%) tested positive. Among the 62 samples (24.12%) without detectable anti-PaBV antibodies, 36 (58.06%) tested negative for anti-ganglioside antibodies and 26 (41.94%) tested positive. The longitudinal variability observed in individual birds (Figure 5) highlights that anti-ganglioside antibodies lack reliability as diagnostic markers for avian ganglioneuritis.

Only 15/33 (45.45%) of samples from birds with clinical signs consistent with avian ganglioneuritis at the time of sampling tested positive for anti-ganglioside antibodies; 18/33 (54.55%) tested negative. Similarly, 99/224 (41.07%) of samples from birds without clinical signs tested positive for anti-ganglioside antibodies, indicating no association between clinical signs and detection of anti-ganglioside antibodies. These findings are consistent with previous results in Quaker parrots, where immunization with gangliosides induced anti-ganglioside antibodies without clinical disease [42].

However, correlation with pathological and histopathological findings is most essential as it is postulated that anti-ganglioside antibodies correlate with ganglioneuritis. In total, 89 samples obtained at or shortly before euthanasia were analyzed. Of these, 25/89 (28.09%) showed proventricular dilatation. Anti-ganglioside antibodies were detected in only 9/25 (36%) of these samples. Because dilatation of the proventriculus is considered a hallmark lesion of avian ganglioneuritis and alternative causes were excluded, these findings indicate that anti-ganglioside antibody testing lacks sufficient diagnostic reliability when correlated with clinical and pathological findings.

No association was found between anti-ganglioside antibody detection and histopathologically confirmed ganglioneuritis. Among birds without mononuclear inflammatory lesions, 14/33 (42.42%) had detectable anti-ganglioside antibodies, whereas 19/33 (57.58%) tested negative. In birds with histopathological lesions consistent with avian ganglioneuritis, 53.57% tested negative, while 46.43% tested positive for anti-ganglioside antibodies. Similarly, 36.36% of birds with mild lesions and 40% with severe lesions were positive, indicating no correlation between antibody detection and lesion severity. Overall, no association was observed between anti-ganglioside antibody detection and clinical, gross pathological, or histopathological findings.

These findings indicate that anti-ganglioside antibody detection does not correlate with the presence of PaBV-associated ganglioneuritis or related clinical signs and are in strong contradiction to previously published findings which indicate a 98% concordance between confirmed avian ganglioneuritis and anti-ganglioside antibodies [40,41]. Preanalytical factors (e.g., storage or transport) may have affected sample quality; however, the uniform distribution of positive results across all groups makes this unlikely. Limitations of the assay must also be considered. Thus, while these results do not exclude a role of anti-ganglioside antibodies in disease pathogenesis, they indicate that their detection lacks sufficient diagnostic reliability [46,47]. As samples were analyzed by a commercial laboratory, the assay reflects current diagnostic practice. Consequently, it can be concluded that a reliable test system for detecting anti-ganglioside antibodies in association with avian ganglioneuritis appears not to be available currently. These results do not provide further evidence that pathogens other than PaBV contribute to the occurrence of avian ganglioneuritis. Proposed differential diagnoses of ganglioneuritis like yeast infections (e.g., *Candida albicans*, *Macrorhabdus ornithogaster*), bacterial infections (e.g., *Escherichia coli*), parasitic infections, heavy metal intoxication (zinc, lead), and other viral infections (e.g., *Psittacine Herpesvirus 1*, *Paramyxovirus*, *West Nile virus*) [45,48,49] were all excluded by pre-study screening, clinical monitoring during the study period, and necropsy including bacteriological, mycological, and parasitological examinations and histopathological investigations. Therefore, proposed other causes for the development of gangioneuritis [36,37] were excluded for all the birds. In addition, no correlation was found between histologically confirmed avian ganglioneuritis and the presence of anti-ganglioside antibodies, further arguing against these alternative causes.

Whether anti-ganglioside antibodies contribute to the development of ganglioneuritis remains unclear and was beyond the scope of this study. Further research is required to clarify their role and to assess the potential involvement of pathogens other than PaBV.

Piepenbring et al. [16,17] fulfilled Koch’s postulates, and multiple infection trials support these findings [13,14,15,21,23,26,50,51]. PaBV remains, to date, the only confirmed cause of avian ganglioneuritis, formerly known as PDD (proventricular dilatation disease). However, detection of PaBV RNA or antibodies in clinically affected birds indicates only a potential cause and does not prove causation; alternative diagnoses must therefore be excluded.

## 5. Conclusions

The results of this study demonstrate that commercially available assays for anti-ganglioside antibodies are not suitable for the diagnosis of avian ganglioneuritis. However, a role of these antibodies in disease pathogenesis cannot be excluded due to potential limitations of the test system and limitations of this study. Further research is required to clarify their role. Until further evidence is available, parrot bornavirus (PaBV) remains the only confirmed cause of avian ganglioneuritis (formerly PDD).

## Figures and Tables

**Table 1:** From the studies of Piepenbring et al. and Gartner et al., 1–6 samples per bird were included in this study. From the studies of Heckmann et al., only samples collected at the time of euthanasia were included because none of the birds seroconverted or showed clinical signs of avian ganglioneuritis.

Study	Piepenbring et al. [17]	Piepenbring et al. [16]	Heckmann et al. [20]	Heckmann et al. [25]	Gartner et al. [34]	Total Amount
During trial	50	38	0	0	80	168
End of trial/death	19	17	14	17	22	89
Total	69	55	14	17	102	257

**Table 2:** Titer grouping of anti-PaBV antibodies (titers <1:10 = group 0, 1:10–1:80 = group 1, 1:160–1:640 = group 2, 1:1280–1:2560 = group 3, and >1:2560 = group 4) and anti-ganglioside antibodies (<160 = negative [group 0], up to 180 = “developing titer” [group 1], up to 220 = “mild clinical disease” [group 2], >220 = “chronic disease” [group 3], and >315 = “advanced clinical disease” [group 4]).

	Anti-PaBV Antibodies					
Anti-Ganglioside Antibodies (Absorptance 405 nm)	0 (<1:10)	1 (1:10–1:80)	2 (1:160–1:640)	3 (1:1280–1:2560)	4 (>1:2560)	Total
<160 (0)	36	13	24	20	57	150
~180 (1)	5	5	6	5	17	38
~220 (2)	15	3	3	5	23	49
>220 (3)	6	0	0	1	12	19
>315 (4)	0	0	0	0	1	1
Total	62	21	33	31	110	257

## Data Availability

Data are contained within the article.
